# Preservation of Involved Teeth Associated with Large Dentigerous Cysts

**DOI:** 10.1155/2014/289463

**Published:** 2014-10-28

**Authors:** Yeliz Guven, Yelda Kasimoglu, Merva Soluk Tekkesin, Dicle Ulug, Abdulkadir Burak Cankaya, Elif Bahar Tuna, Koray Gencay, Oya Aktoren

**Affiliations:** ^1^Department of Pedodontics, Faculty of Dentistry, Istanbul University, Capa, 34093 Istanbul, Turkey; ^2^Department of Tumour Pathology, Institute of Oncology, Istanbul University, 34093 Istanbul, Turkey; ^3^Department of Oral Surgery, Faculty of Dentistry, Istanbul University, 34093 Istanbul, Turkey

## Abstract

Dentigerous cysts (DCs) are benign odontogenic cysts that are associated with the crowns of permanent teeth. The purpose of this study is to describe the management of DCs in four children. Four boys aged between 7 and 9 years were referred to our clinics with the complaints of intraoral alveolar swelling or facial asymmetry on the affected area. The panoramic radiographies showed large, well-defined radiolucent lesions associated with the deciduous teeth and displaced tooth buds. The treatment consisted of the extraction of the involved deciduous tooth and marsupialization of the cyst to allow eruption of the permanent tooth. Permanent teeth displaced by the DCs in three cases erupted spontaneously within one-year period. The case with horizontally displaced permanent tooth was managed by replantation. This is the first time that underlying permanent tooth in a DC case was intentionally replanted.

## 1. Introduction

Dentigerous cysts (DC) are benign odontogenic lesions caused by an alteration of reduced enamel epithelium which results in fluid accumulation either between the reduced enamel epithelium and the crown of an unerupted/impacted tooth or within the enamel organ itself. DCs are usually asymptomatic, and they may be detected incidentally via routine radiographic examination or when they are large enough (>2 cm in diameter) to cause facial asymmetry [1–3]. Clinical signs suggestive of a DC include a retained deciduous tooth, delayed eruption of a permanent tooth, and painless swelling of the involved area. In radiographs, the DC appears as a well-defined unilocular radiolucency associated with the crown of an unerupted tooth. Often the radiolucent area surrounds the crown, but sometimes it lies mainly or entirely to one side [4, 5].

Different theories have been proposed to explain the etiology of the DC. The first theory suggests that DCs are developmental in origin and occur in mature teeth usually as a result of impaction. These cysts usually occur in the late second and third decades, predominantly involving mandibular third molars. The second theory advocates that DCs are inflammatory and occur in immature teeth as a result of inflammation from a nonvital deciduous tooth or another source spreading to involve the tooth follicle. These are diagnosed in the first and early part of second decade, predominantly involving mandibular premolars [6].

It is important to emphasize that DCs in children might show a rapid and painless expansion and may result in fractures and deformation of facial bone structures. Early diagnosis and treatment are therefore important. The aim of the present paper is to describe clinical, radiological, and histopathological findings of four additional cases with DCs treated by conservative surgical techniques. To our knowledge, this study constitutes the first reported case of DC in which affected permanent tooth was intentionally replanted to its place after marsupialization.

## 2. Subjects and Methods

**Figure 1:**
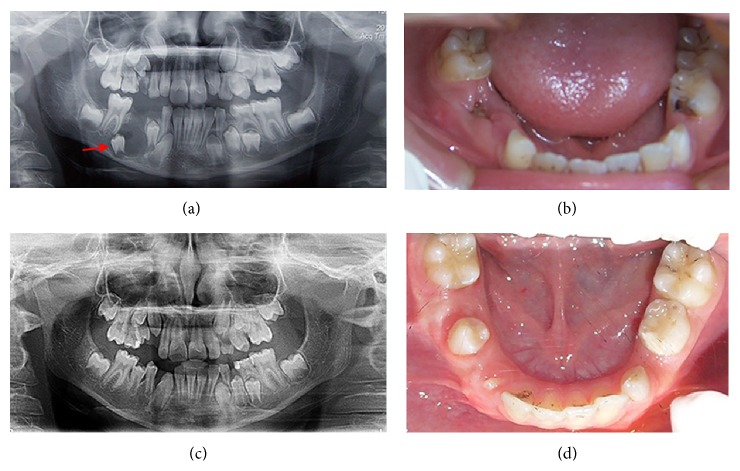
Case 1: an 8-year-old boy. (a) Initial panoramic X-ray image showing a DC in the right mandibular region. (b) Intraoral view of the cystic region. Tooth 85 had been extracted 1 week before the initial examination. (c)-(d) 16-month follow-up X-ray image and intraoral photos showing the complete healing of the cystic region and eruption of the affected tooth.

**Figure 2:**
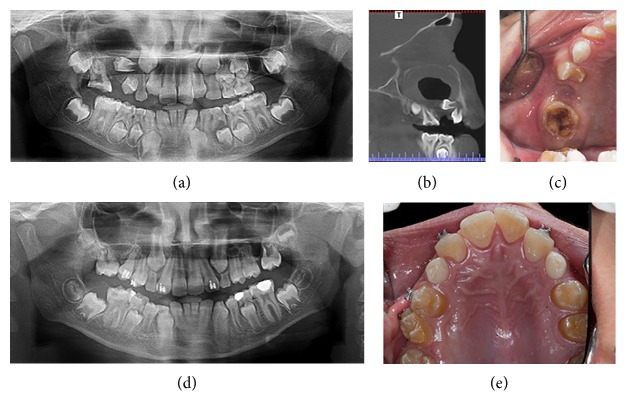
Case 2. (a)-(b) Initial panoramic X-ray image (a) and coronal CT image (b) indicating a DC in the right maxillary region. (c) Initial intraoral view. (d)-(e) 27-month follow-up panoramic radiograph and intraoral image showing the complete healing of the cystic region.

**Figure 3:**
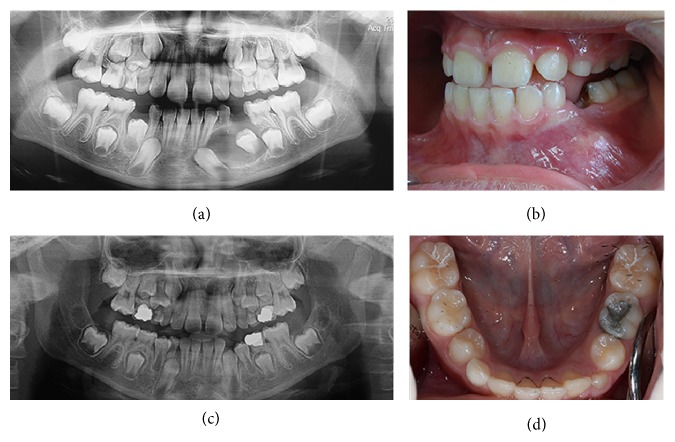
Case 3. (a) Initial panoramic X-ray image showing a DC in the left mandibular region. (b) Initial intraoral view of the cystic region. (c)-(d) 12-month follow-up X-ray image and intraoral photo showing the complete healing of the cystic region and eruption of the affected tooth.

**Figure 4:**
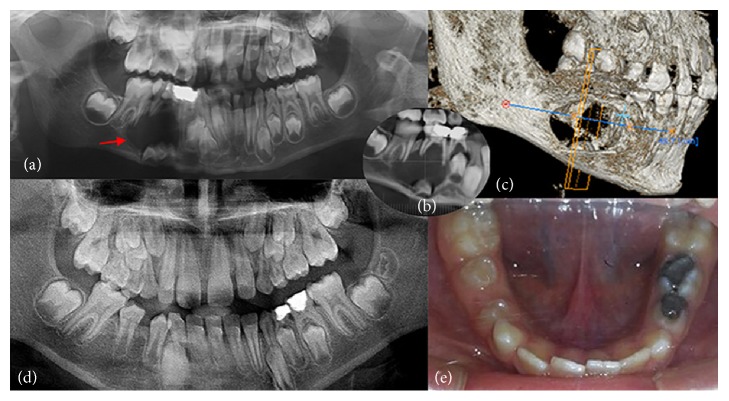
Case 4. (a)–(c) Initial panoramic X-ray image (a) and CBCT images (b)-(c) indicating a large DC in the right maxillary region. (d)-(e) 26-month follow-up X-ray image and intraoral photo showing the complete healing of the cystic region and eruption of the affected tooth.

Four boys, aged between 7 and 9 years, were referred to clinics in the Department of Pediatric Dentistry at Istanbul University with the chief complaints of intraoral alveolar swelling or facial asymmetry on the affected area. The patients were evaluated by a comprehensive clinical and radiologic examination, including panoramic radiography and cone beam computed tomography (CBCT). Clinical and radiographic images of these cases are shown in Figures 1, 2, 3, and 4.

All of the cases were treated by extraction of the deciduous tooth around the cyst and marsupialization of the cyst to allow eruption of the permanent tooth. In all cases, a biopsy tissue sample was taken from the access window of marsupialization for the histopathological examination, and then a silicon tube was installed to drain the intracystic pressure. The histopathologic examination of the lesions confirmed the diagnostic hypothesis of a dentigerous cyst with inflammatory origin.

**Figure 5:**
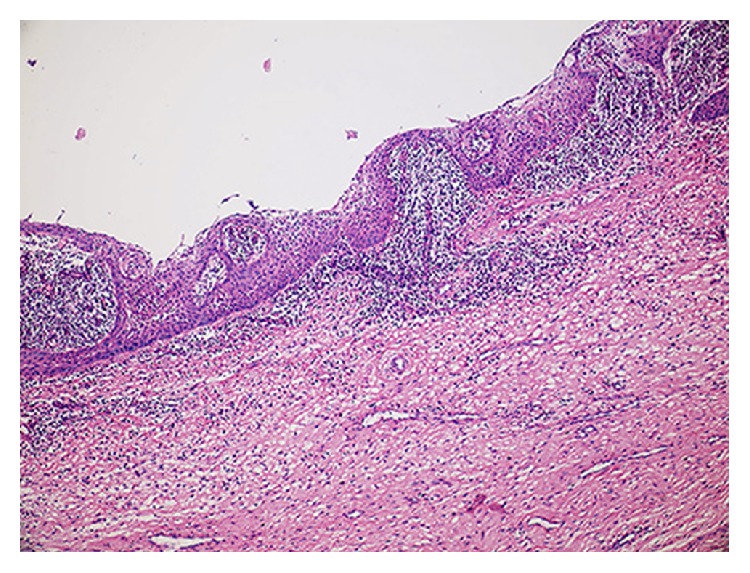
The cyst was lined by nonkeratinized, flat, stratified squamous epithelium. Note the high inflammatory infiltrate in the fibrous wall (H&E, ×100).

**Figure 6:**
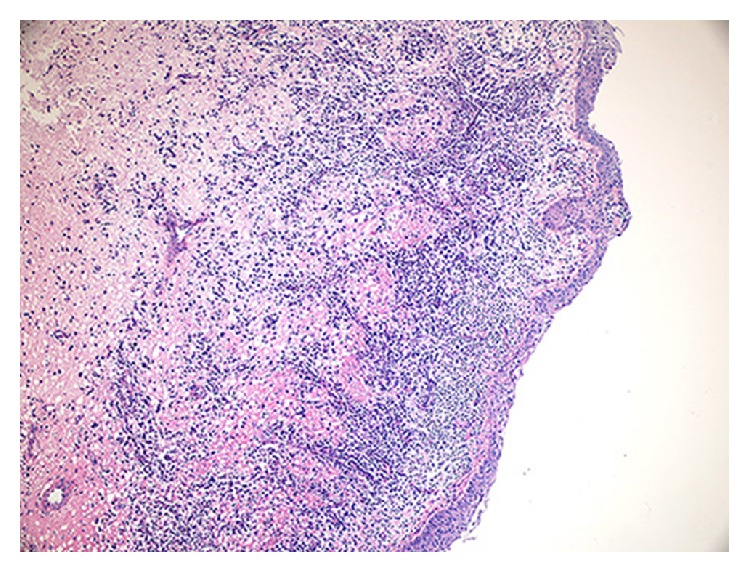
The epithelial lining of inflamed dentigerous cyst was thick with hyperplastic rete ridges. The connective tissue wall showed a diffuse chronic inflammatory infiltrate (H&E, ×100).

In our study, the histopathologic features of all four cases were similar. Figures 5 and 6 show representative pictures of the cysts, all of which were inflamed. The cysts were lined by stratified squamous epithelium which demonstrated hyperplastic rete ridges, particularly in high inflamed areas. The epithelium-connective tissue junction was flat in some areas. The fibrous wall of the cysts contained an inflammatory infiltrate containing lymphocytes variably intermixed with plasma cells and eosinophils.

Permanent teeth displaced by the DCs in three cases erupted in proper position spontaneously within a one-year period. The other case with a horizontally displaced permanent premolar did not have a chance to erupt spontaneously; thus intentional replantation was chosen as the treatment. After nine months of healing of the cyst cavity, the tooth was extracted and replaced in its proper place in the arch by surgery in a position where the alveolar bone support is highest. Therefore, the crown of the replanted tooth was in a slightly vestibular position at the end of surgery. The position of the tooth was corrected orthodontically. A follow-up visit at twenty-eight months showed complete healing of the surrounding bone and the tooth was asymptomatic.

## 3. Discussion

DCs are the third most common odontogenic cysts, after radicular cysts and odontogenic keratocyst, and account for approximately 10.39% of all odontogenic cysts [7]. Patients with DCs have no painful symptoms unless acute inflammatory exacerbation is present. Swelling can be experienced if the DC grows too large or lies in a sensitive area. Koca et al. evaluated thirty-five children diagnosed with DC and determined that swelling was the main complaint in 70% of the children, while 5% experienced pain and 25% had no symptoms [8]. In the present cases, the main complaint of two patients (Cases 2 and 4) was facial swelling, while the main complaint of Case 1 was a hard swelling described around the buccal sulcus of the premolar region. Case 3 was referred to our faculty when no regression could be provided with a one-week IM antibiotic treatment on swelling.

Considering that the size of a normal follicular space in radiographs is 3 to 4 mm in diameter, a DC can be suspected when a follicular space measuring more than 5 mm is observed [9, 10]. Conventional panoramic and periapical radiographs are sufficient to detect the lesion, but they may fail to delineate the full extent of the lesion. It is therefore important to use more advanced imaging techniques, such as 3D CT, especially in extensive lesions. CT imaging provides more accurate information about the lesion's size, its relationship to adjacent anatomical structures, and the position of the underlying permanent tooth [11]. It is emphasized that an additional CT scan should be preferred for large lesions extending to the nasal cavity or orbital or pterygomaxillary space [8]. In the present study, an additional CT scan was performed only in Cases 2 and 4. A CT scan in Case 2 was required for the complete delineation of the cystic lesion considering its close proximity to the maxillary sinus and the horizontal position of the involved premolar. In Case 4, the cystic lesion was so extensive and close to the base of the mandible that the CT scan was helpful in assessing the precise extent of the lesion. In all cases, panoramic radiographs were preferred for the periodic follow-up visits.

Radicular cysts described in association with the pulpotomized primary molars seem to show similar clinical features with inflammatory dentigerous cysts. Several differences were reported between the radiological features of radicular cysts and dentigerous cysts by various reports [11–13]. Basically, the pericoronal space of the underlying permanent tooth in radicular cysts appears normal, usually with a total or partially distinct cortical layer of bone. DCs, on the other hand, generally have no distinct boundaries between the roots of primary tooth and the crown of the underlying permanent teeth [13]. Even though these differences are not adequate for a definitive diagnosis, they are still important for a provisional diagnosis. Shaw et al. noted that distinguishing inflammatory dentigerous and radicular cysts on histologic grounds is also difficult [14]. Therefore, it is important to examine clinical, radiological, and histological findings together to make a definite diagnosis. Because DCs have the potential to form an ameloblastoma, an accurate diagnosis and its differentiation from a radicular cyst are of paramount importance [15]. In the present study, a provisional diagnosis of DC was made for all of the cases, based on clinical and radiographical examination. Histopathological examination confirmed the diagnosis and revealed that all of the cases were inflammatory in origin.

DCs are treated via surgical enucleation of the cystic lesion or marsupialization technique. Enucleation involves the complete removal of all tissues involved with the cyst, including removal of the unerupted tooth. Marsupialization or decompression is a technique that attempts to relieve intracystic pressure through the creation of an accessory cavity [1]. The treatment approach for children with DCs should be less invasive to preserve affected permanent teeth, as teeth with open apices have great eruptive potential. DCs in growing children are mostly treated by marsupialization. In the present cases, marsupialization was the treatment of choice because of the young ages of the patients. Although the cystic lesions had changed the position of the underlying permanent teeth in varying degrees, all teeth except Case 2 returned to a satisfactory position and erupted spontaneously after treatment. In Case 2, the horizontally displaced maxillary second premolar was unlikely to erupt on its own. Therefore, after maintaining the complete healing of the cystic lesion and considerable amount of bone was formed at about nine months, the tooth was replanted to its correct position by another surgical operation.

The major disadvantage of marsupialization is pathologic tissue left in situ without a thorough histological examination, because of the possibility that a more aggressive lesion may occur in the residual tissue. However, recurrence of DC is rare, especially after complete removal of the cyst or tooth eruption [16]. In the present cases, conservative treatment led to a high quality outcome. All teeth maintained their vitality and no recurrence was observed at the follow-up periods.

Conservative treatment is a favorable treatment modality for DCs in growing children and adolescents. Considering that the regeneration capacity of the bony structures in children is greater than in adults and the teeth with open apices have greater eruptive potential, efforts should be made to allow the involved tooth to erupt spontaneously. Intentional replantation could also be employed in teeth which have a great strategic value for maintaining the balanced occlusion. It is important to follow up with patients regularly for the possibility of recurrence.
